# Supplementary material for: Splice-Junction-Based Mapping of Alternative Isoforms in the Human Proteome
Source: Cell Rep. Author manuscript; Available in PMC 2020 Jan 15. (PMC6961840; doi:10.1016/j.celrep.2019.11.026)

A

sp|P29692|EF1D_HUMAN|ENSG00000104529|RI1|5616|chr8|143581154|143581328|−2|r368|T4,sp|P29692|EF1D_HU
VGTDLLEEEITKFEEHVQSVDIAAFNKL q value: 1.726e−05 Tr_novel:TRUE RefSeq_Novel:TRUE
Search result spec prec mz: 794.6649 Actual spec prec mz: 794.66492
Fragments matched per AA: 2.61 Proportion of top 20 peaks matched: 0.45

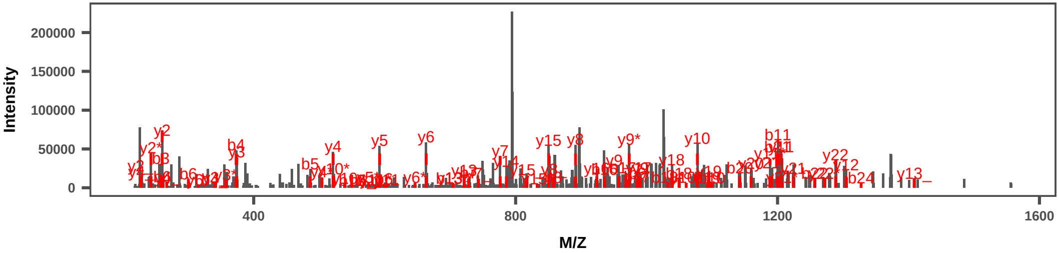

B

Scatterplot of predicted elution time
Fitting R2: 0.859
Novel peptide residual Z score: 0.861
Number of peptides: 1340

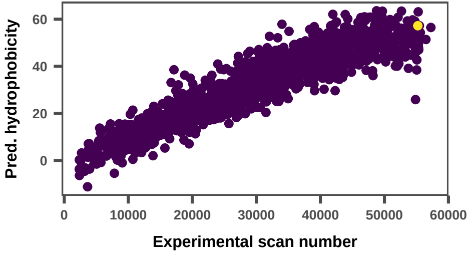

C

Distributions of residuals from best−fit line
of predicted RT vs Expt. scan number
Line: Z score of novel peptide
Z: 0.861

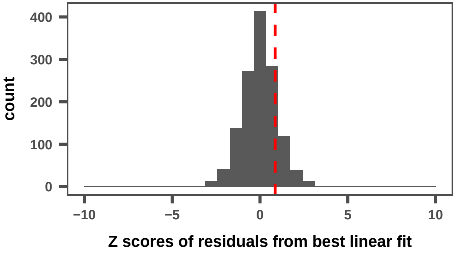

Supplement: 2 [file NIHMS1546469-supplement-2.zip › DF1/PXD010154/Lung/Lung_3_EEF1D_VGTDLLEEEITKFEEHVQSVDIAAFNKL.pdf]
